# Methodological Literacy as an Ethical Competence in Clinical Practice: A Conceptual Argument From Dentistry

**DOI:** 10.1111/jep.70494

**Published:** 2026-06-07

**Authors:** Clovis Mariano Faggion

**Affiliations:** ^1^ Department of Periodontology and Operative Dentistry, Faculty of Dentistry University Hospital Münster Münster Germany

**Keywords:** critical appraisal, dental education, ethics, evidence‐based dentistry, methodological literacy, research methodology, shared decision‐making

## 1 Introduction

Ethics occupies a central place in dentistry, shaping responsibilities toward patients, colleagues, and society. Beyond technical competence, ethical obligations include evaluating scientific evidence and integrating it with patient values and clinical expertise. Core principles—autonomy, beneficence, non‐maleficence, and justice—are operationalized through informed consent, professional codes, and clinical guidelines [1, 2].

Dentistry is increasingly recognised as an evidence‐based profession, with growing emphasis on the explicit use and critical appraisal of scientific evidence in clinical decision‐making [3]. Yet, ethical discourse and evidence‐based practice are sometimes treated as parallel rather than interdependent domains [4]. This separation can compromise clinical and ethical quality, particularly when assessing new materials, techniques, or protocols with limited evidence.

While evidence‐based dentistry (EBD) is widely endorsed, methodological competence is typically taught as a brief component of broader EBD instruction rather than as a longitudinal professional competency. This raises a central concern: current educational approaches may not adequately prepare dentists to interpret and communicate scientific evidence ethically in real clinical settings. This discussion does not critique existing ethical principles outlined in guidelines such as the World Dental Federation (FDI) but proposes that contemporary practice requires supplementing these principles with explicit epistemic competencies.

In this article, methodological literacy refers to the set of competencies that enable clinicians to critically evaluate the validity, reliability, and applicability of clinical research and incorporate these judgements into patient‐centred decision‐making under conditions of uncertainty. Although closely related to evidence‐based practice, methodological literacy has rarely been conceptualised as a distinct educational or professional competency and is used here to describe competencies associated with methodological appraisal and the communication of uncertainty in clinical care [5, 6]. It comprises three interrelated components: (i) interpretation of study design and methodological validity, (ii) appraisal of risk of bias, effect estimates, and certainty of evidence, and (iii) communication of benefits, harms, and uncertainty within shared decision‐making (SDM).

It extends general EBD competence by moving beyond question formulation and literature searching toward epistemic evaluation and its ethical application. It is also distinct from clinical judgement alone, as it requires explicit engagement with methodological quality and limits of evidence rather than reliance on experiential knowledge.

This commentary argues that current ethical and educational frameworks insufficiently specify how methodological understanding should inform clinical decision‐making, that this gap has ethically relevant consequences under conditions of uncertainty and innovation, and that methodological literacy should therefore be reframed as an ethical rather than purely technical competence.

## 2 The Problem of Vagueness in Ethical and Educational Guidance

Ethical guidelines, including the FDI Dental Ethics Manual [2], emphasise professional values such as competence, compassion, autonomy, and respect for patients, alongside lifelong learning and continuing professional development (CPD). However, guidance on maintaining and updating competence is often general, leaving methodological skills underemphasised.

In dentistry, CPD largely evaluates courses on practical methods, procedures, and technologies, with limited evidence on impacts on professional practice or critical application of evidence‐based approaches [7]. For instance, while courses introducing new implant or restorative systems often emphasise procedural steps and hands‐on training, they rarely require structured engagement with the underlying evidence base, including study design limitations, risk of bias, certainty of outcomes and applicability to individual patients. This creates a gap between technical adoption and epistemic justification, particularly when evidence is preliminary or derived from small, industry‐supported studies. Dedicated courses on research methodology, critical evaluation, and uncertainty remain rare.

At the undergraduate dental level, research methodology and critical appraisal are usually embedded within broader EBD courses rather than taught as longitudinal, standalone competencies. Instruction tends to emphasise formulating clinical questions and conducting literature searches, while giving comparatively less attention to deeper methodological concepts.

For example, students may be exposed to published trials showing statistically significant improvements of a new dental material, yet receive limited training in interpreting whether these effects are clinically meaningful, generalisable, or robust against bias. Similarly, they may encounter systematic reviews with heterogeneous or low‐certainty evidence without structured guidance on how such uncertainty should influence clinical decision‐making. In several curricula identified in a scoping review [8], EBD teaching was concentrated in the first and second years of dental education. This timing may limit opportunities for students to progressively integrate methodological understanding with later clinical experience and decision‐making. These considerations suggest that methodological literacy should be developed progressively across all years of study.

## 3 Methodological Literacy as an Ethical Requirement

Methodological literacy encompasses competencies related to evaluating how evidence is produced, including recognition of threats to validity and interpretation of effect estimates in context within clinical decision‐making [9].

It complements traditional clinical competence by enabling clinicians to judge likely benefits and harms accurately and to provide patients with clear, evidence‐based information, including uncertainties, supporting informed participation in decisions [10].

Insufficient methodological literacy has direct ethical consequences in clinical practice. Weak appraisal of evidence may lead to inappropriate clinical decisions, including overinterpretation of findings, premature adoption of interventions, or overtreatment, as well as failure to recognise meaningful benefits. It may also limit clinicians' ability to appropriately contextualise evidence and its uncertainty, contributing to suboptimal communication and formally adequate but substantively weak informed consent. As a result, clinical recommendations may not be fully aligned with the best available evidence, and patient involvement in decision‐making may be compromised. In fact, new dental technologies or materials may enter practice based on findings that may not accurately reflect clinical reality. For example, in periodontology and implant dentistry, early trials have been shown to report larger treatment effects than later studies, a phenomenon sometimes referred to as 'novelty bias', with subsequent evidence often demonstrating smaller or more uncertain benefits [11]. Apparent effects may also reflect limited follow‐up, methodological constraints, or statistical and methodological heterogeneity [12]. Conversely, interventions with modest statistical effects may still be clinically important depending on baseline risk, absolute benefit, or patient preferences. Such competencies are therefore required to distinguish statistical signals from clinically relevant outcomes and to avoid both overinterpretation and underestimation of evidence.

Contemporary ethical guidance emphasises the importance of lifelong competence, but often does not explicitly address methodological competence. Because clinical decisions involve judgements made under conditions of uncertainty, epistemic responsibilities—such as appropriately interpreting evidence quality, recognising uncertainty, and avoiding overstatement of findings—acquire ethical significance through their effects on patient autonomy, informed consent, and the risk–benefit balance of care [13]. Methodological literacy therefore represents an integral element of responsible clinical practice. Because patients are generally unable to independently evaluate the methodological quality and limitations of clinical evidence, clinicians occupy a fiduciary and epistemically privileged position that creates an ethical responsibility to interpret and communicate evidence competently and transparently. This capacity underpins SDM, as collaboration with patients is meaningful only when clinicians can interpret and clearly communicate evidence, particularly when it is uncertain, conflicting, or of low quality [14]. Without these skills, informed consent risks becoming a procedural formality rather than a substantive ethical process. This concern is especially relevant as patients increasingly access independent health information that requires careful interpretation and contextualisation [15].

Recognising the limitations of evidence also fosters epistemic humility, which supports cautious and transparent clinical reasoning when evidence is incomplete or evolving [13]. In clinical practice, this translates into proportionate communication of uncertainty and appropriately calibrated adoption of interventions. This is particularly important in dentistry, where frequent innovation in materials, devices, and techniques challenges clinicians to critically evaluate emerging evidence. By integrating patient perspectives into this appraisal, clinicians support equitable, responsible, and patient‐centred care [16].

## 4 Implications for Dental Education and Professional Guidance

If methodological literacy is an ethical requirement, education must develop the capacity to interpret and communicate evidence under uncertainty.

At the undergraduate level, methodological competence could be embedded into clinical teaching through structured ‘evidence‐in‐action’ formats, where students are required to appraise one clinical decision per case using primary research, systematic reviews, and clinical guidelines, explicitly linking study quality, effect size, and clinical applicability to treatment planning, and supported by interactive or flipped learning strategies that promote active engagement, self‐directed preparation, and in‐class problem‐solving, although evidence on their long‐term impact remains limited [17]; assessment could move beyond written examinations to include objective structured clinical examinations with stations that evaluate interpretation of uncertainty, risk of bias, and communication of evidence to simulated patients.

At the postgraduate and CPD level, training could shift from primarily procedural certification toward structured, evidence‐critical learning. This may include mandatory critical appraisal sessions or journal clubs linked to real clinical cases, where dentists are required to justify decisions based on the strength, limitations, and applicability of available evidence rather than merely summarising publications. Competence could be assessed through portfolio‐based evaluation documenting how methodological reasoning informs clinical decisions over time. Similarly, activities within CPD could move beyond passive attendance at technique‐oriented workshops toward accreditation, requiring participants to evaluate methodological principles and the external validity of the interventions, technologies, or guidelines being introduced.

These strategies are complementary rather than stage‐specific and may be adapted across undergraduate, postgraduate, and continuing education to support longitudinal development of methodological literacy. These strategies should be understood as proposed directions for curricular development rather than established educational interventions, as current evidence on their long‐term effectiveness in dental education remains limited.

## 5 Conclusion

Ethical dentistry extends beyond codes, guidelines, or technical standards. In an evidence‐rich but uncertain environment, ethical practice requires methodological literacy. Dentists lacking this competence may risk undermining informed consent, SDM, and patient trust.

Recognising methodological literacy as an ethical requirement promotes reflective, transparent, and patient‐centred practice. Future revisions of ethical guidelines and educational frameworks should explicitly link ethics with epistemic responsibility. Incorporating case scenarios in education and policy guidance helps clinicians navigate complex contemporary practice, with relevance extending beyond dentistry.

## Funding

The author has nothing to report.

## Ethics Statement

This article does not contain any studies involving human participants or animals performed by the author.

## Conflicts of Interest

The author declares no conflicts of interest.

## Data Availability

Data sharing not applicable to this article as no datasets were generated or analysed during the current study.

## References

[1] B. Varkey , “Principles of Clinical Ethics and Their Application to Practice,” Medical Principles and Practice 30, no. 1 (2021): 17–28, 10.1159/000509119. 32498071 PMC7923912

[2] Dental Ethics Manual | FDI, accessed April 22, 2026, https://www.fdiworlddental.org/resource/dental-ethics-manual.

[3] Evidence‐Based Dentistry (EBD) | FDI, accessed February 5, 2026, https://www.fdiworlddental.org/evidence-based-dentistry-ebd.

[4] Upshur REG ., “A Call to Integrate Ethics and Evidence‐Based Medicine,” Virtual Mentor: VM 15, no. 1 (2013): 86–89, 10.1001/virtualmentor.2013.15.1.oped1-1301. 23356814

[5] M. Hultcrantz , D. Rind , E. A. Akl , et al., “The GRADE Working Group Clarifies the Construct of Certainty of Evidence,” Journal of Clinical Epidemiology 87 (2017): 4–13, 10.1016/j.jclinepi.2017.05.006. 28529184 PMC6542664

[6] J. P. T. Higgins , D. G. Altman , P. C. Gotzsche , et al., “The Cochrane Collaboration's Tool for Assessing Risk of Bias in Randomised Trials,” BMJ 343 (2011): d5928, 10.1136/bmj.d5928. 22008217 PMC3196245

[7] V. R. Firmstone , K. M. Elley , M. T. Skrybant , A. Fry‐Smith , S. Bayliss , and C. J. Torgerson , “Systematic Review of the Effectiveness of Continuing Dental Professional Development on Learning, Behavior, or Patient Outcomes,” Journal of Dental Education 77, no. 3 (2013): 300–315. 23486894

[8] D. Qin , X. Wu , F. Li , X. Yu , and F. Hua , “Teaching Critical Appraisal and Research Methodology to Dental Students and Practitioners: A Scoping Review,” Journal of Dentistry 160 (2025): 105930, 10.1016/j.jdent.2025.105930. 40555303

[9] K. Galbraith , A. Ward , and C. Heneghan , “A Real‐World Approach to Evidence‐Based Medicine in General Practice: A Competency Framework Derived From a Systematic Review and Delphi Process,” BMC Medical Education 17, no. 1 (2017): 78, 10.1186/s12909-017-0916-1. 28468646 PMC5415750

[10] F. Asa'ad , “Shared Decision‐Making (SDM) in Dentistry: A Concise Narrative Review,” Journal of Evaluation in Clinical Practice 25, no. 6 (2019): 1088–1093, 10.1111/jep.13129. 30920092

[11] M. C. Menne , G. Seitidis , C. M. Faggion Jr , D. Mavridis , and N. Pandis , “Early Optimistic Effect in Periodontology and Implant Dentistry Trials,” Journal of Dental Research 101, no. 1 (2022): 30–36, 10.1177/00220345211025242. 34237225 PMC8721552

[12] C. M. Faggion Jr , M. A. Atieh , M. Tsagris , J. Seehra , and N. Pandis , “A Case Study Evaluating the Effect of Clustering, Publication Bias, and Heterogeneity on the Meta‐Analysis Estimates in Implant Dentistry,” European Journal of Oral Sciences 132, no. 1 (2024): e12962, 10.1111/eos.12962. 38030576

[13] A. Schwab , “Epistemic Humility and Medical Practice: Translating Epistemic Categories Into Ethical Obligations,” Journal of Medicine and Philosophy 37, no. 1 (2012): 28–48, 10.1093/jmp/jhr054. 22241866

[14] T. C. Hoffmann , V. M. Montori , and C. Del Mar , “The Connection Between Evidence‐Based Medicine and Shared Decision Making,” Journal of the American Medical Association 312, no. 13 (2014): 1295–1296, 10.1001/jama.2014.10186. 25268434

[15] P. Shah , I. Thornton , N. L. Kopitnik , and J. E. Hipskind , “Informed Consent.” StatPearls (StatPearls Publishing, 2025), http://www.ncbi.nlm.nih.gov/books/NBK430827/. 28613577

[16] D. Z. Buchman , A. Ho , and D. S. Goldberg , “Investigating Trust, Expertise, and Epistemic Injustice in Chronic Pain,” Journal of Bioethical Inquiry 14, no. 1 (2017): 31–42, 10.1007/s11673-016-9761-x. 28005251

[17] O. Al‐Karadsheh , H. Abutayyem , A. Saidi , and A. Shqaidef , “Knowledge Acquisition and Student Perceptions of Three Teaching Methods: A Randomized Trial of Live, Flipped, and Interactive Flipped Classrooms,” BMC Medical Education 25, no. 1 (2025): 573, 10.1186/s12909-025-07156-0. 40251621 PMC12008932

